# Enhanced osteogenesis of human urine-derived stem cells by direct delivery of 30Kc19α–Lin28A protein

**DOI:** 10.3389/fbioe.2023.1215087

**Published:** 2023-06-13

**Authors:** Jinhee Park, Kiho Jeong, Manho Kim, Wijin Kim, Ju Hyun Park

**Affiliations:** Department of Biomedical Science, Kangwon National University, Chuncheon-si, Gangwon-do, Republic of Korea

**Keywords:** LIN28A, 30Kc19α, protein delivery, urine-derived stem cells, osteogenesis

## Abstract

Urine-derived stem cells (USCs) are a promising source for regenerative medicine because of their advantages such as easy and non-invasive collection from the human body, stable expansion, and the potential to differentiate into multiple lineages, including osteoblasts. In this study, we propose a strategy to enhance the osteogenic potential of human USCs using Lin28A, a transcription factor that inhibits let-7 miRNA processing. To address concerns regarding the safety of foreign gene integration and potential risk of tumorigenicity, we intracellularly delivered Lin28A as a recombinant protein fused with a cell-penetrating and protein-stabilizing protein, 30Kc19α. 30Kc19α–Lin28A fusion protein exhibited improved thermal stability and was delivered into USCs without significant cytotoxicity. 30Kc19α–Lin28A treatment elevated calcium deposition and upregulated several osteoblast-specific gene expressions in USCs derived from multiple donors. Our results indicate that intracellularly delivered 30Kc19α–Lin28A enhances the osteoblastic differentiation of human USCs by affecting the transcriptional regulatory network involved in metabolic reprogramming and stem cell potency. Therefore, 30Kc19α–Lin28A may provide a technical advancement toward developing clinically feasible strategies for bone regeneration.

## 1 Introduction

Somatic stem cells (SSCs) found in adult tissues differentiate into various cell types and have been extensively studied for their potential use in tissue regeneration. However, obtaining SSCs from patient tissue requires specialized medical personnel and invasive procedures that expose the patient to pathogens. Consequently, obtaining a sufficient number of cells can be challenging (Robertson, 2001). Adipose-derived stem cells (ADSCs) have been widely studied for tissue regeneration. However, obtaining ADSCs from patients through liposuction can result in cosmetic issues due to skin incisions (Bellini et al., 2017). Bone marrow-derived mesenchymal stem cell (BM-MSC) extraction, also requires an invasive procedure (García Quiroz et al., 2008). In contrast, human urine-derived stem cells (USCs) can be obtained from patients safely and non-invasively. They are also more telomere-active and have longer telomeres than BM-MSCs, allowing for mass culture (Shi et al., 2012). Approximately 2,000–7,000 human USCs are excreted in urine daily, which can be cultured *in vitro* (Rahmoune et al., 2005). The phenotype of USCs is similar to that of MSCs (Bharadwaj et al., 2011), and USCs differentiate into various cell types, including osteoblasts, chondrocytes, and adipocytes (Zhang et al., 2008; Wu et al., 2011; Bharadwaj et al., 2013; Guan et al., 2014).

USCs possess a greater potential for differentiation into myogenic, neurotrophic, and endothelial lineages than ADSCs (Kang et al., 2015). Since they originate from the upper urinary tract (Zhang et al., 2008), their differentiation potential is similar to that of mesodermal MSCs and ectoderm epithelial stem cells. Epithelial stem cells are primarily located in hair follicles and have ectodermal potency (Ohyama, 2007). They can easily differentiate into neurons and smooth muscle cells; however, their potential for differentiation into bone, cartilage, and fat cells is lower than that of ADSCs and BM-MSCs (Kang et al., 2015; Sun et al., 2021). Therefore, to use USCs as alternative SSCs, their potential for differentiation into adipocytes, osteoblasts, and chondrocytes must be improved.

Lin28A and its paralog, Lin28B, are RNA-binding proteins that heterochronically regulate mRNA translation. Both homologs selectively suppress let-7 miRNA expression, thereby preventing let-7 miRNA-mediated embryonic stem cell (ESC) differentiation and contributing to the pluripotent state maintenance (Martinez and Gregory, 2010; Piskounova et al., 2011). Lin28A plays a crucial role in regulating the early stages of embryonic development and organ formation (Moss et al., 1997; Yokoyama et al., 2008). The mode of action of Lin28A in embryonic cells involves enhanced glucose metabolism and ATP generation (Shyh-Chang and Daley, 2013). Previous studies have shown that Lin28A enhances aerobic glycolysis by activating pyruvate dehydrogenase kinase 1 (PDK1), and its overexpression reprograms cell metabolism for ATP production from an oxidative phosphorylation (OxPhos)-dominant phenotype to a more glycolytic state, even in an aerobic environment (Ma et al., 2014; Docherty et al., 2016). In *postpartum* tissues, Lin28A overexpression accelerated tissue recovery and improved glucose tolerance in a mouse model (Shyh-Chang et al., 2013). Although the exact mechanism remains unclear, the enhanced glycolytic metabolism of Lin28A-expressing cells and the resulting low mitochondrial metabolic characteristics have been suggested to reduce oxidative stress that can occur during OxPhos, thereby promoting tissue regeneration. Lin28A-expressing SSCs with fused and healthier mitochondrial phenotypes exhibit enhanced self-renewal and differentiation capacities into specific lineages such as osteoblasts and chondrocytes, demonstrating that reduced oxidative damage to mitochondria plays a key role in Lin28A-mediated tissue regeneration (Pieknell et al., 2022).

This study aimed to enhance the osteogenic differentiation of human USCs using Lin28A as a recombinant protein. Lin28A was delivered intracellularly by fusing it with the N-terminal α-helix domain of the silkworm-derived 30Kc19 protein, known as 30Kc19α. Previous studies have demonstrated that fusion with 30Kc19 delivers non-permeable proteins in various mammalian cells and improves the stability of fusion partner proteins (Lee et al., 2019; Lee J et al., 2020; Kim et al., 2022; Son et al., 2022). Additionally, 30Kc19α retains the cell-penetrating and protein-stabilizing properties of the full-length 30Kc19 (Ryu et al., 2016; Lee H et al., 2020; Lee et al., 2022). Our results indicate that the 30Kc19α–Lin28A fusion protein can be delivered into USCs without any significant cytotoxicity, increasing their osteogenic differentiation. This approach offers a new perspective on the use of cell-penetrating proteins to enhance the therapeutic capabilities of human USCs without the need for transgenes, thus paving the way for a clinically feasible strategy in regenerative medicine.

## 2 Materials and methods

### 2.1 Isolation and culture of human USCs

The USCs were isolated from 250 to 300 mL urine samples collected from three healthy male donors aged 32–42 years by centrifugation at 500 *g* for 10 min. The cell pellets were washed with phosphate-buffered saline (PBS) supplemented with 100 μg/mL primocin (InvivoGen, San Diego, CA, United States) and then resuspended in primary medium comprising DMEM/F-12 (Thermo Fisher Scientific, Waltham, MA, United States), 10% fetal bovine serum (FBS, Welgene, Daejeon, Korea), renal epithelial growth medium (REGM) SingleQuots Kit (Lonza, Basel, Switzerland), and 100 μg/mL primocin. The cells were plated on 0.2% gelatin-coated six-well culture plates and incubated for 3 days. The cells were then cultured in growth medium, which comprised 1:1 REGM (basal media supplemented with 10% FBS, REGM SingleQuots Kit, and 100 μg/mL primocin) and mesenchymal cell proliferation medium (high glucose DMEM supplemented with 10% FBS, 1× GlutaMax, 1× non-essential amino acids [Thermo Fisher Scientific], 5 ng/mL basic fibroblast growth factor [bFGF; PeproTech, Seoul, Korea], 5 ng/mL platelet-derived growth factor-AB [PDGF-AB, PeproTech], and 5 ng/mL epithelial growth factor [EGF; R&D Systems, Minneapolis, MN, United States]). Upon reaching 80% confluence, the USCs were detached using 0.25% trypsin-EDTA (Thermo Fisher Scientific) and reseeded on gelatin-coated plates. Human osteoblasts (OBs) (#CC-2538; Lonza) were cultured in osteoblast growth medium (PromoCell, Heidelberg, Germany). Human dermal fibroblasts (HDFs) and MCF-7 cells, a human breast cancer cell line, were cultured in DMEM supplemented with 10% FBS and 1% penicillin–streptomycin (Thermo Fisher Scientific).

### 2.2 Expression and purification of recombinant proteins

The cDNA sequences of Lin28A obtained from the pCXLE-hUL plasmid (Addgene plasmid #27080) and the 30Kc19α used in the previous study (Ryu et al., 2016) were cloned into the pET-23a vector to bind Lin28A to the C-terminus of 30Kc19α. Exponentially growing cultures of *Escherichia coli* transformed with the recombinant vector were treated with 0.5 mM isopropyl-β-D-thiogalactopyranoside at 25°C for 16 h. After cell lysis by sonication, the soluble fraction of the recombinant protein was purified using Ni-NTA chromatography (HisTrap HP, Cytiva, Uppsala, Sweden). After dialysis against DMEM, the purified protein samples were mixed with a reducing sample buffer containing sodium dodecyl sulfate (SDS) and β-mercaptoethanol and loaded on a 10% polyacrylamide gel for separation. Protein bands were visualized through Coomassie Brilliant Blue R-250 (Sigma-Aldrich, St. Louis, MI, United States) staining.

### 2.3 Immunostaining analysis and cytotoxicity test

For immunostaining, the cells were fixed with 4% paraformaldehyde (PFA) and permeabilized with 0.25% Triton X-100 in PBS. After blocking, cells were sequentially incubated with anti-T7 tag polyclonal primary antibody (Abcam, Cambridge, United Kingdom) at 4°C overnight and Alexa Fluor 488-conjugated secondary antibody (Thermo Fisher Scientific) at room temperature for 1 h. After treatment with 4′,6-diamidino-2-phenylindole (DAPI, Sigma-Aldrich) to stain the cell nuclei, fluorescence images were obtained by confocal laser scanning microscopy (CarlZeiss, Oberkochen, Germany). To investigate the cytotoxicity of 30Kc19α–Lin28A protein treatment, USCs (2×10^4^ cells/cm^2^) were seeded on 0.2% gelatin-coated 96-well plates and incubated for 24 h in growth medium. After treatment with 30Kc19α–Lin28A in growth medium for 24 h, a water-soluble tetrazolium (WST)-8 solution (Quanti-MAX WST-8 Cell Viability Assay Kit reagent; Biomax, Seoul, Korea) was added to each well and incubated for another 2 h. Finally, absorbance was measured at 460 nm using a microplate reader.

### 2.4 *In vitro* osteogenesis of human USC

To induce osteoblast differentiation, human USCs (2×10^4^ cells/cm^2^) were seeded in a 0.2% gelatin-coated 24-well plate and incubated for 24 h in growth medium. The growth medium was then replaced with DMEM supplemented with 10% FBS, 10 mM-glycerol phosphate, 50 μg/mL ascorbic acid, and 100 nM dexamethasone (osteogenic medium), and the cells were further cultured with medium changes every 2 days. During the first week of the osteoblast differentiation, the cells were treated two or three times with 30Kc19α–Lin28A, with a two-day interval between each treatment. Subsequently, the culture was continued in osteogenic medium.

### 2.5 Alizarin Red S staining and OsteoImage mineralization assay

For Alizarin Red S (ARS) staining, the differentiated cells were fixed with 4% PFA and washed thrice with PBS. Then, they were stained with 2% ARS solution (Sigma-Aldrich) as previously described (Lee J et al., 2020) and observed under a microscope. Briefly, each stained culture was incubated with 10% acetic acid and gently shaken for 10 min at room temperature. After heating at 85°C for 10 min and neutralizing with 10% ammonium hydroxide, the absorbance at 405 nm was measured using a microplate reader. The calcified matrices were visualized using the OsteoImage mineralization assay according to the manufacturer’s instructions (Lonza). After fixation with 70% ethanol for 20 min, a fluorescent staining reagent was added to each well and incubated for 30 min in dark. The bone-like calcified matrices were observed using a fluorescence microscope.

### 2.6 Reverse transcription polymerase chain reaction

Total RNA was isolated using the Ribospin Total RNA Purification Kit (GeneAll Biotechnology Co., Ltd., Seoul, Korea) according to the manufacturer’s instructions. The isolated RNA was then reverse transcribed into cDNA using TOPScript RT DryMIX (Enzynomics Co., Ltd.) and the dT 18 plus primer. For reverse transcription polymerase chain reaction (RT-PCR) analysis, each cDNA was amplified using 2× TOPsimple DyeMIX-HOT (Enzynomics Co. Ltd.) and relevant primers in a T100 Thermal Cycler (Bio-Rad, Hercules, CA, United States) for 25 cycles. Quantitative real-time PCR (qPCR) was performed using TOPreal qPCR 2× PreMIX (SYBR Green with low ROX; Enzynomics) with glyceraldehyde 3-phosphate dehydrogenase serving as an internal control. All experiments were conducted in quadruplicate, and the relative comparison between cell groups was determined using the 2^−ΔΔCT^ method. The primer sequences used for both RT-PCR and qPCR analyses are summarized in Supplementary Table S1.

### 2.7 Transcriptome analysis

For RNA-sequencing (RNA-seq) analysis, total RNA was isolated using the TRI reagent (Thermo Fisher Scientific), and its quality was evaluated using the Agilent TapeStation 4000 system (Agilent Technologies, Amstelveen, Netherlands). The RNA library of the control and experimental groups were constructed by employing the QuantSeq 3′mRNA-Seq Library Prep Kit (Lexogen Inc., Vienna, Austria) according to the manufacturer’s instructions. After amplifying the library amplification to include the complete adapter sequences required for cluster generation, the finished library was purified from the PCR components. High-throughput single-end sequencing was performed using NextSeq 550 (Illumina, Inc., United States) as single-end 75 sequencing. Gene classification was based on searches conducted using DAVID (http://david.abcc.ncifcrf.gov/) and Medline (http://www.ncbi.nlm.nih.gov/). Raw data and graphic visualization were processed using ExDEGA graphic software (Ebiogen, Seoul, Korea). Genes with ≥1.5 fold change (upregulated/downregulated) were considered statistically significant. The STRING software (https://string-db.org/) was used to evaluate possible interactions, co-expressed genes, and protein networks.

### 2.8 Statistical analysis

All results are expressed as mean ± standard deviation. Statistical significance was confirmed using Student’s t-test and one-way analysis of variance (ANOVA). The results were considered significant at *p* < 0.05. All quantitative analyses presented in this study were obtained from replicate samples in a representative experiment conducted several times.

## 3 Results and discussion

### 3.1 Characterization of donor-specific USCs

**FIGURE 1:**
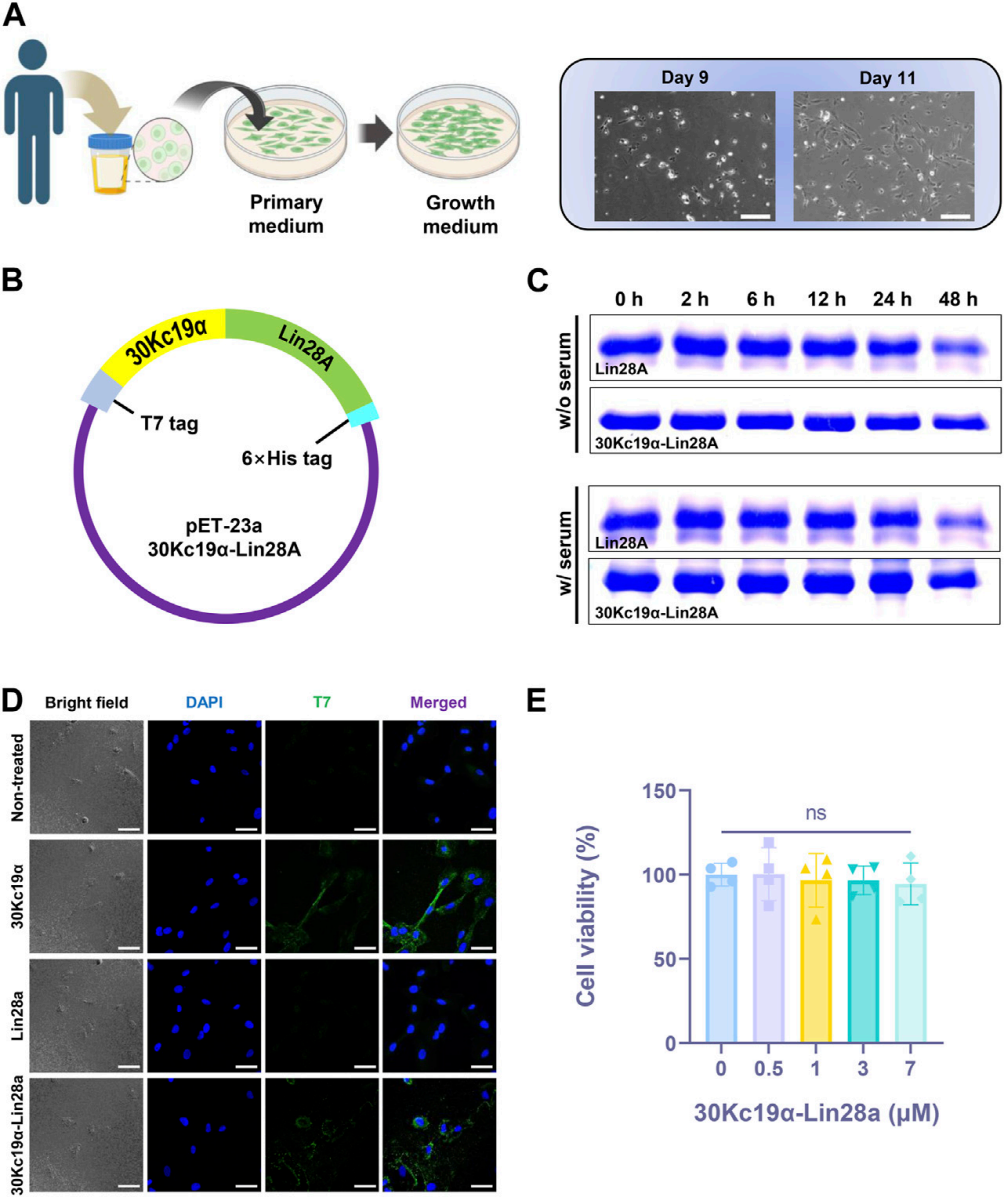
Expression of 30Kc19α–Lin28A protein from *Escherichia coli* system and characterization of its physiological properties. **(A)** A schematic illustration of the process of isolating urine-derived stem cells (USCs) from a urine sample (created with BioRender.com), and images of the cultured USCs. Scale bar = 200 µm. **(B)** *E. coli* expression vector construction. **(C)** Thermal stability of Lin28A and 30Kc19α–Lin28A analyzed by sodium dodecyl sulfate polyacrylamide gel electrophoresis (SDS–PAGE) after incubation in serum-containing and serum-free DMEM for up to 48 h at 37 °C. **(D)** Confocal laser scanning microscopy images of USCs treated with 2 µM 30Kc19α, Lin28A, and 30Kc19α–Lin28A for 2 h. Intracellularly delivered protein was detected using an anti-T7 tag polyclonal antibody and an Alexa 488-conjugated secondary antibody (green). The cell nuclei were stained with 4′,6-diamidino-2-phenylindole (DAPI; blue). Scale bars = 40 µm. **(E)** Viability of USCs evaluated by water-soluble tetrazolium (WST)-8 assay after treatment with up to 7 µM 30Kc19α–Lin28A for 24 h. The error bars indicate the standard deviation (*n* = 4), ns, not significant.

USCs were isolated from urine samples obtained from three donors. Initially, each isolated USC was cultured in the primary medium and then switched to the growth medium. Microscopic observation revealed that the cultured cells exhibited a fibroblast-like morphology, which is consistent with previously reported characteristics (Figure 1A). As previous studies have reported that human USCs share similar characteristics with MSCs (Bharadwaj et al., 2011; Bharadwaj et al., 2013; He et al., 2016), we investigated the expression of the typical MSC surface markers CD73, CD90, and CD105 in isolated human USCs (Supplementary Figure S1). Despite some variations in the expression levels, RT-PCR analysis demonstrated that all three USC lines expressed these positive MSC marker mRNAs. These markers were detected in human BM-MSCs, but not in MCF-7 cells, indicating successful USC isolation from the three different donors.

### 3.2 Effects of 30Kc19α fusion on Lin28A stability and cell-permeability

The 30Kc19α–Lin28A fusion protein was designed to link Lin28A to the C-terminus of 30Kc19α. All recombinant proteins used in this study had a T7 tag at the N-terminus for immunostaining, and a 6× histidine tag at the C-terminus for Ni-NTA affinity chromatography (Figure 1B). SDS–PAGE indicated that the molecular weights of purified proteins corresponded to their theoretical sizes (13, 26, and 39 kDa for 30Kc19α, Lin28A, and 30Kc19α–Lin28A, respectively; Supplementary Figure S2). We investigated whether 30Kc19α fusion could enhance the stability of Lin28A by comparing the degradation rate at high temperatures. After incubation at 37°C in serum-free and 10% FBS-containing DMEM for different time intervals, the remaining Lin28A and 30Kc19α–Lin28A were analyzed by SDS–PAGE (Figure 1C). The amount of non-degraded Lin28A significantly reduced after 48 h of incubation, whereas 30Kc19α–Lin28A remained more stable than intact Lin28A. These results indicate that 30Kc19α fusion can improve Lin28A stability in the culture environment.

To examine the effect of 30Kc19α fusion on intracellular delivery, we randomly selected a particular cell line, designated USC #1, from a group of three independent cell lines. Subsequently, the cells were treated with equimolar amounts of 30Kc19α, Lin29A, and 30Kc19α–Lin28A for 2 h. Confocal microscopy revealed that 30Kc19α and 30Kc19α–Lin28A were delivered intracellularly, whereas Lin28A did not penetrate the cell significantly (Figure 1D). Subsequently, the cytotoxicity of the 30Kc19α–Lin28A protein was analyzed by WST-8 assay. The USCs were treated with various 30Kc19α–Lin28A concentrations of for 24 h in proliferative medium. Even at the highest concentration (7 μM), the cell viability did not decrease significantly (Figure 1E). These results clearly indicate that 30Kc19α fusion confers a cell-penetrating property to the non-permeable Lin28A protein without any significant cytotoxicity.

### 3.3 30Kc19α–Lin28A promotes osteogenesis in USCs

A decline in the self-renewal and differentiation potential of SSCs is a well-known hallmark of aging (Roobrouck et al., 2008). Previous studies have demonstrated that forced Lin28A expression in various SSCs via lentiviruses can increase cell expansion and proliferative marker expression. Additionally, Lin28A overexpression improves bone and cartilage differentiation of aged MSCs, thereby enhancing the tissue repair capacity of SSCs after transplantation (Pieknell et al., 2022). Despite its benefits, the lentiviral delivery of Lin28A raises safety concerns for clinical applications, including tumorigenesis and alterations in cell fate due to non-specific chromosomal modifications in recipient SSCs. To overcome these limitations of lentivirus-mediated DNA delivery, various integration-free strategies, such as RNA and protein delivery, have been developed. However, direct protein delivery requires repeated protein transduction owing to low stability and limited intracellular protein delivery, which can lead to severe cytotoxicity. Based on the results presented in Figure 1, which confirmed the high stability, intracellular delivery, and low cytotoxicity of the 30Kc19α–Lin28A protein, we investigated its potential on the osteoblast differentiation of USCs.

**FIGURE 2:**
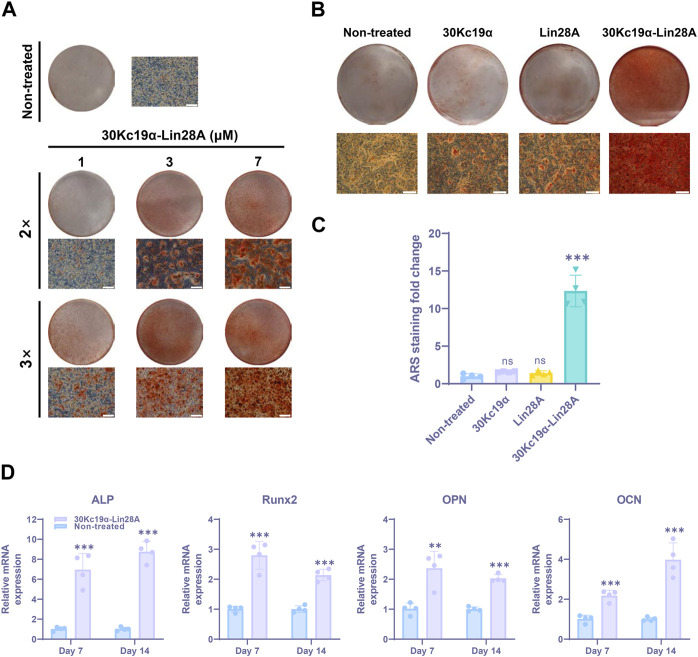
Effect of 30Kc19α–Lin28A on urine-derived stem cell (USC) osteogenesis (USC #1). **(A)** Alizarin red S (ARS) staining images showing calcium deposition in USCs after osteogenic culture for 21 days. The cells were treated with up to 7 µM 30Kc19α–Lin28A two or three times during the first week of osteogenesis. **(B)** ARS staining images of USCs treated with 30Kc19α, Lin28A, and 30Kc19α–Lin28A. After treatment with 7 µM protein three times, the cells were further cultured in osteogenic medium. On day 21, ARS staining was performed and **(C)** quantitatively analyzed. The error bars indicate the standard deviation (*n* = 4), ***p < 0.005, ns: not significant compared to the non-treated control. Scale bar = 100 µm. **(D)** Gene expression of osteoblast-specific markers (ALP, Runx2, OPN, OCN). The mRNA expression level of each gene was determined using quantitative real-time PCR. mRNA samples were obtained from USCs treated with 30Kc19α–Lin28A on day 14 of osteogenic culture. The error bars indicate the standard deviation (*n* = 4), **p < 0.01, ***p < 0.005 compared to the non-treated control.

Since Lin28A is associated with inhibiting the terminal differentiation of SSCs (Molenaar et al., 2012; Urbach et al., 2014), excessive exposure to Lin28A might interfere with USC differentiation. Therefore, we aimed to determine the optimal condition for 30Kc19α–Lin28A treatment. Human USCs (USC #1) were treated with 0–7 μM 30Kc19α–Lin28A protein two or three times during the early stage of osteogenesis (Supplementary Figure S3). ARS staining revealed that 30Kc19α–Lin28A dose-dependently enhanced USC osteogenesis. At the highest dose (7 μM, three times), ARS-stained calcium deposits covered most of the culture area, whereas no significant staining was observed in the non-treated control cells. In contrast to that with two treatments with 7 μM 30Kc19α–Lin28A, osteogenesis was significantly enhanced with the third treatment (Figure 2A). The OsteoImage mineralization assay also demonstrated the enhanced osteogenesis of USCs by 30Kc19α–Lin28A, showing that the calcium deposits increased than that in the non-treated control (Supplementary Figure S4). Accordingly, we subsequently treated USCs with 7 μM 30Kc19α–Lin28A three times during the first week of osteogenesis. Following three treatments with 30Kc19α-Lin28A, there were no notable differences in cell confluence during the continuous osteogenesis process, indicating that the proliferation of USCs was not significantly affected by the treatment with 30Kc19α-Lin28A. We also assessed the effects of 30Kc19α, Lin28A, and 30Kc19α–Lin28A proteins on USC osteogenesis. The cells were treated with 7 μM each protein three times, and calcified matrix formation was evaluated after osteogenic induction for 21 days. ARS staining revealed no significant effect of 30Kc19α and Lin28A on USC osteogenesis, whereas calcified matrices substantially covering the entire culture area were formed in only 30Kc19α–Lin28A-treated cells (Figure 2B). Quantitative analysis for the ARS staining also demonstrated that 30Kc19α–Lin28A treatments enhanced the osteoblastic differentiation of USCs, in contrast to 30Kc19α or Lin28A alone (Figure 2C). Considering the results of the cell penetration test, we postulated that this result could be attributed to the inability of Lin28A to penetrate the cells, whereas 30Kc19α penetrated the cells but did not enhance osteogenesis. These findings support our conclusion that Lin28A delivered intracellularly by fusion with the cell-penetrating 30Kc19α, enhances the differentiation potential of USCs into osteoblasts. While there is a possibility that the conjugated 30Kc19α could have a minor influence on the osteogenesis-promoting effect of 30Kc19α-Lin28A, it is more reasonable to attribute the primary functionality to the Lin28A itself.

To further investigate the impact of 30Kc19α–Lin28A on the osteogenesis of USCs, we examined the expression levels of osteoblast marker genes through qRT-PCR analysis. Specifically, the mRNA expression levels of the osteoblast-specific markers runt-related transcription factor 2 (Runx2), alkaline phosphatase (ALP), osteopontin (OPN), and osteocalcin (OCN) were compared on days 7 and 14 of osteogenesis. Our results indicated that 30Kc19α–Lin28A treatment significantly increased the mRNA expressions of both early (Runx2 and ALP) and late (OPN and OCN) osteogenic markers (Figure 2D). Although this increase in expression varied slightly for each marker with differentiation duration, 30Kc19α–Lin28A treatment clearly upregulated the expression of osteoblast-related genes, thereby enhancing the osteogenesis of human USCs.

### 3.4 Comparison of differentially expressed genes (DEGs) in 30Kc19α–Lin28A-treated USCs

**FIGURE 3:**
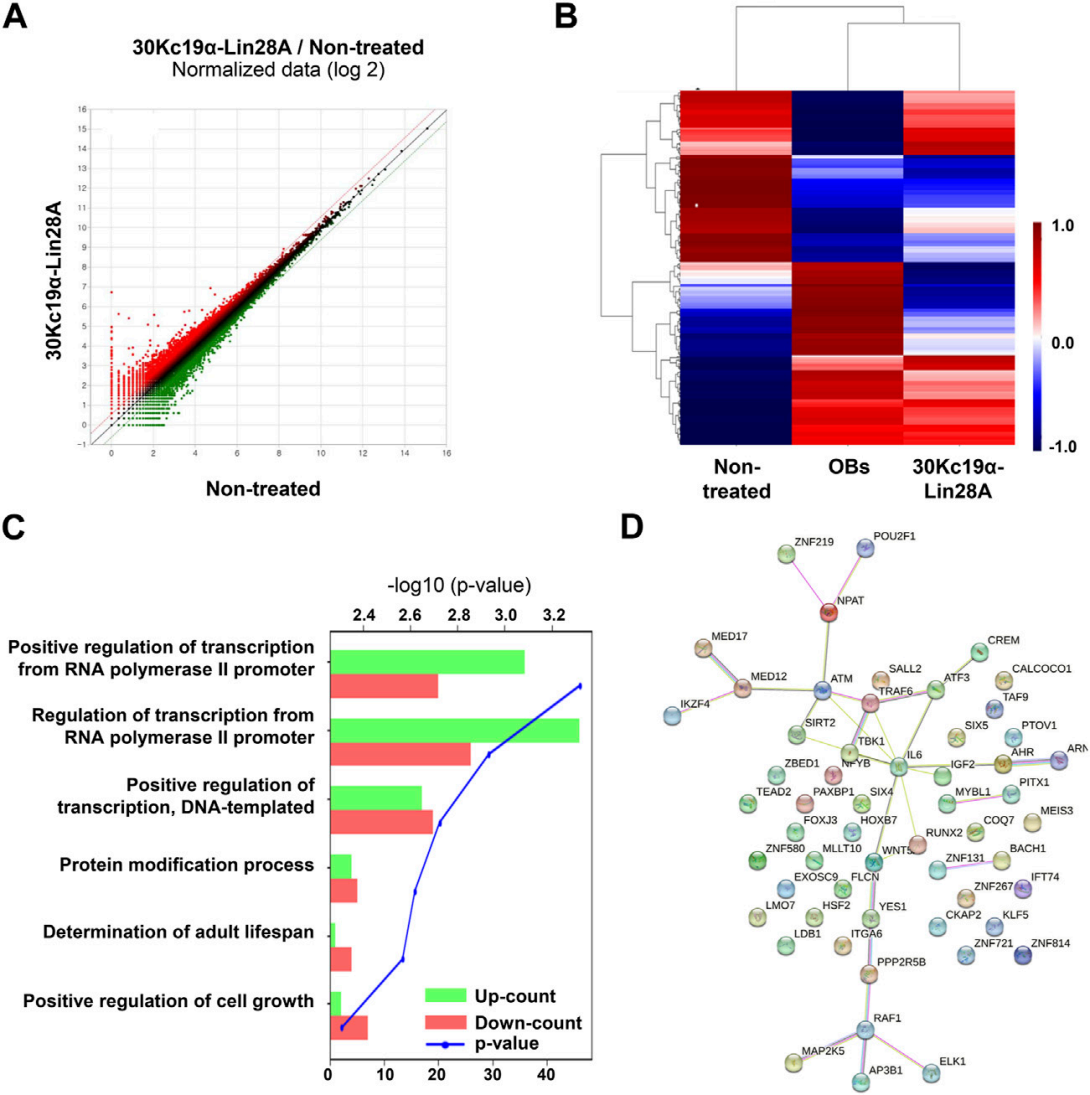
Transcriptomic analysis of urine-derived stem cells (USCs; USC #1). Total RNA was extracted from non-treated and 30Kc19α–Lin28A-treated USCs on day 14 of osteogenic culture and subjected to RNA-seq analysis. **(A)** Scatter plot showing the differentially expressed genes (DEGs) in 30Kc19α–Lin28A-treated USCs compared to those in non-treated USCs **(B)** Hierarchical clustering heatmap analysis of the DEGs in 30Kc19α–Lin28A-treated USCs and osteoblasts (OBs) compared to that in USCs cultured in growth medium. **(C)** Gene ontology analysis of upregulated and downregulated genes in 30Kc19α–Lin28A-treated USCs compared to non-treated USCs. **(D)** STRING analysis of upregulated DEGs.

To evaluate the changes in transcriptional profiles, the RNA of 30Kc19α–Lin28A-treated human USCs, non-treated control cells, and OBs were extracted after 14 days of osteogenesis and compared. The scatter plot analysis of the identified transcripts revealed 648 DEGs with 1.5-fold change in 30Kc19α–Lin28A-treated USCs compared to that in non-treated control cells (Figure 3A). A heat map obtained by hierarchical clustering of DEGs showed that the 30Kc19α–Lin28A-treated group and OBs clustered together and were separated from non-treated control cells, indicating that 30Kc19α–Lin28A treatment enhanced osteogenesis (Figure 3B).

Several genes, including Raf1 and Wnt5A involved in cell proliferation, differentiation, survival, and osteogenic differentiation, were upregulated. Previous studies have reported that Raf1 plays a pivotal role in preventing the apoptosis of osteocytes induced by fatigue loading (Li et al., 2011), and Wnt5A-induced noncanonical Wnt signaling promotes MSC osteogenesis and suppresses adipogenesis (Takada et al., 2007; Takada et al., 2009). Conversely, among the downregulated genes, PPP2R5B is a subunit of PP2A that regulates the cell cycle and growth factor signaling. The reduction of PP2A expression promotes osteogenic differentiation by regulating bone-forming transcription factors, such as osterix (Okamura et al., 2017).

Gene ontology analysis revealed a significantly altered expression of genes involved in transcriptional regulation by the RNA polymerase II promoter (Figure 3C). Previous studies have shown that phosphorylated extracellular signal-related kinase (p-ERK) phosphorylates Runx2, which is necessary for inducing osteoblast-specific transcription (Ge et al., 2009). This p-ERK-mediated Runx2 phosphorylation initiates a series of chromatin changes associated with RNA polymerase II recruitment and transcription. RNA polymerase II recruitment is also related to the functions of several homeodomain proteins, such as Dlx3 and Dlx5, which play crucial roles in the transcriptional regulation of osteoblast differentiation. These homeodomain proteins, along with Runx2 and OCN, are coordinately recruited with increased occupancy of RNA polymerase II (Hassan et al., 2004; Hassan et al., 2006). These findings suggest that 30Kc19α–Lin28A treatment is likely to promote osteogenesis in USCs by regulating transcriptional processes through RNA polymerase II. Furthermore, a STRING analysis of all DEGs revealed gene clusters involved in osteogenesis, indicating the transcriptomic responses of 30Kc19α–Lin28A-treated USCs toward osteogenesis (Figure 3D).

Despite the transcriptomic changes after 30Kc19α–Lin28A treatment, RNA-seq analysis has some limitations. The analysis of a single sample instead of multiple replicates for each experimental group made it challenging to minimize the variation in the measurement of the expression level of each transcript, even though the analysis of clusters with multiple genes was available. Moreover, there was a lack of detailed analysis regarding the impact of 30Kc19α–Lin28A on the transcriptional regulatory network and cellular signaling that enhanced the osteogenesis of USCs. Conducting large-scale analysis with an increased number of repeated samples in future studies would provide detailed insights into the molecular mechanism through which intracellularly delivered 30Kc19α–Lin28A proteins affect the transcriptional changes in USCs.

### 3.5 Effect of 30Kc19α–Lin28A on USCs of different donors

**FIGURE 4:**
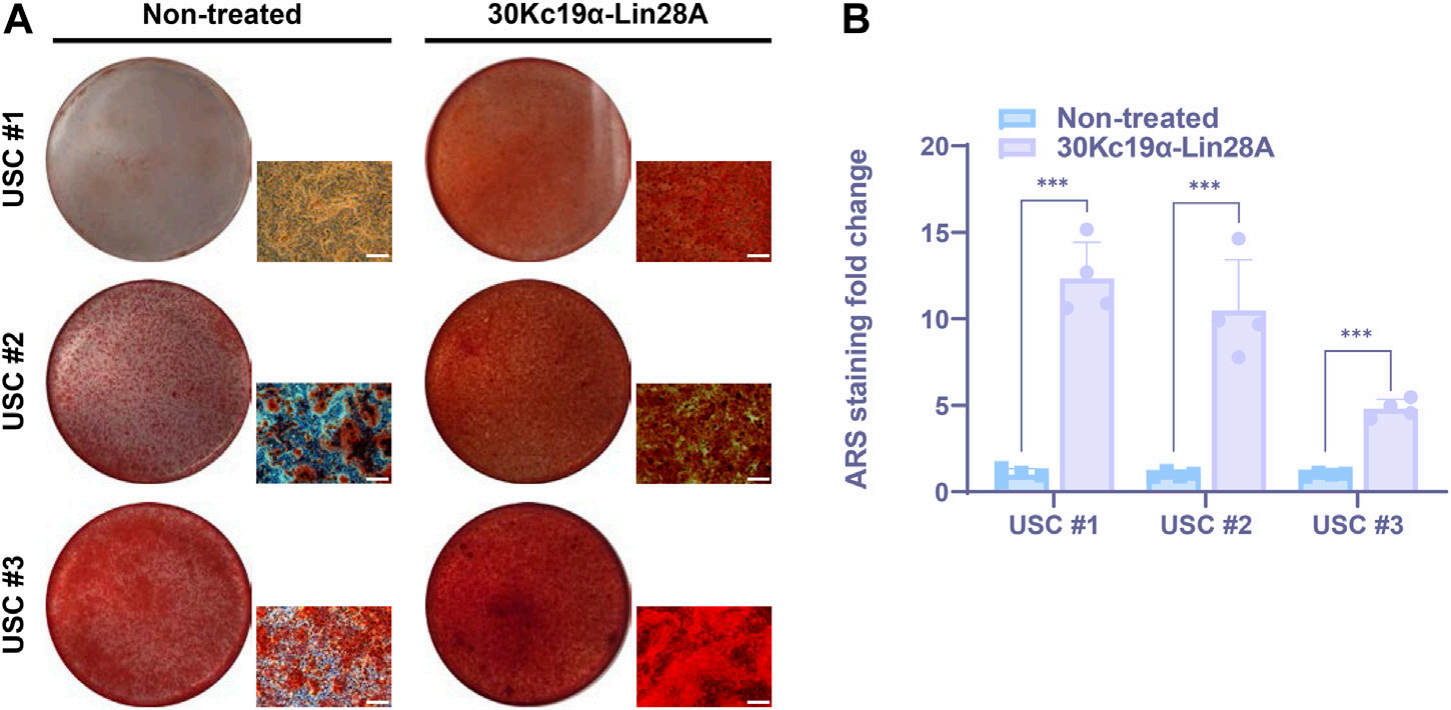
Effect of 30Kc19α–Lin28A on osteogenesis of urine-derived stem cell (USC) lines derived from multiple donors. **(A)** Alizarin red S (ARS) staining images of three USC lines from each independent donor after treatment with 7 µM 30Kc19α–Lin28A three times, followed by osteogenic culture for 21 days. **(B)** Quantitative analysis of ARS staining for calcium deposition. The error bars indicate the standard deviation (*n* = 4), ***p < 0.005. Scale bar = 100 µm.

SSCs display notable variations in their self-renewal and differentiation potentials depending on the donor (Heathman et al., 2016; Zhang et al., 2021). To investigate such differences, we treated three independent USC lines derived from each donor with 30Kc19α–Lin28A protein. After osteoblast differentiation for 21 days, ARS staining revealed a significant variation in ossification levels between the cell lines, indicating donor-specific variation in osteogenic potential. Nevertheless, all 30Kc19α–Lin28A-treated USC lines exhibited excellent calcium deposition (Figure 4A). Interestingly, the osteogenesis of USC #3, which was at least partially differentiated into osteoblasts, in contrast to USC #1 used in previous experiments, was also significantly enhanced by 30Kc19α–Lin28A treatment (Figure 4B). These findings indicate the potential utility of 30Kc19α–Lin28A protein for the clinical applications of USCs obtained from various donors. However, it is widely acknowledged that a notable reduction in the expression of the tumor suppressor let-7 miRNA is a common occurrence in various types of human cancers (Nair et al., 2012). Since the maturation process of the primary let-7 transcript is impeded by the highly conserved RNA-binding protein, Lin28, the oncogenic potential of Lin28A is a major obstacle to clinical application. Nevertheless, in the present study, we administered the 30Kc19α-Lin28A protein to human USCs in three intermittent cycles, followed by osteogenic induction without any further treatment. Compared to previous studies that utilized viral gene delivery for continuous Lin28A expression (Shyh-Chang et al., 2013; Pieknell et al., 2022), the intermittent exposure to Lin28A through protein delivery may substantially reduce the risk of tumorigenicity by minimizing alterations in the transcriptional regulatory network. In addition, based on the results of RNA-seq analysis, when investigating the genes related to tumor cell response, there were no significant differences in expression levels between non-treated and 30Kc19α-Lin28A-treated USCs (Supplementary Figure S5). These results suggest that the administration of 30Kc19α-Lin28a protein had minimal impact on the transcriptional network involved in tumor cell response and tumorigenicity in human USCs.

## 4 Conclusion

Human USCs offer several advantages for cell therapy, including non-invasiveness, easy accessibility, stable culture, high proliferation, differentiation potential toward multiple lineages, and a lack of tumorigenicity. Therefore, they have emerged as promising resources for regenerative medicine (Sun et al., 2021). In this study, we present an alternative approach for enhancing the therapeutic potential of USCs as a source for bone regeneration. Our strategy involved the delivery of Lin28A, a RNA-binding protein that plays a crucial role in metabolic reprogramming, in the form of a recombinant protein. To facilitate intracellular delivery, Lin28A was fused to 30Kc19α, which possesses cell-penetrating and protein-stabilizing properties. We found that 30Kc19α–Lin28A was more stable than intact Lin28A and delivered into USCs without significant cytotoxicity. 30Kc19α–Lin28A treatment promoted the osteogenesis of USCs derived from various donors, as evidenced both phenotypically and genotypically. These findings enable an efficient and safe programming of human USCs into target lineages, including osteoblasts, without the risk of random genetic mutations. We anticipate that our study will facilitate technical advances in the development of therapeutic strategies for bone-related diseases such as osteoporosis.

## Data Availability

The datasets presented in this study can be found in online repositories. The names of the repository/repositories and accession number(s) can be found below: https://www.ncbi.nlm.nih.gov/, GSE233420.
