# Supplementary material for: Enhanced osteogenesis of human urine-derived stem cells by direct delivery of 30Kc19α–Lin28A protein
Source: Front Bioeng Biotechnol. 2023 Jun 13;11:1215087. doi: 10.3389/fbioe.2023.1215087 (PMC10293758; doi:10.3389/fbioe.2023.1215087)
Supplement: Supplementary file 1 [file Table1.DOCX]

Supplementary Material

**Enhanced osteogenesis of human urine-derived stem cells by direct delivery of 30Kc19α-Lin28A protein**

Jinhee Park, Kiho Jeong, Manho Kim, Wijin Kim, and Ju Hyun Park^*^

Department of Biomedical Science, Kangwon National University, Chuncheon-si, Gangwon-do 24341, Republic of Korea

*** Correspondence:** Ju Hyun Park: juhyunpark@kangwon.ac.kr

1. **Supplementary Figures and Tables**

## Supplementary Figures


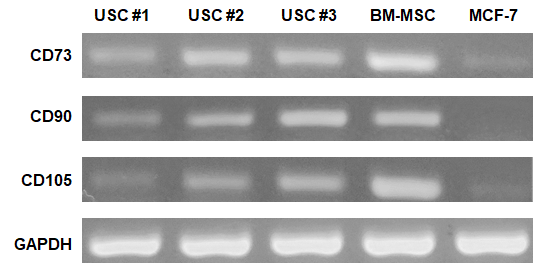


**Supplementary Figure 1.** RT-PCR analysis of human BM-MSC-specific genes (CD73, CD90, CD105) in the isolated USCs from three independent donors. MCF-7 cells, a human breast cancer cell line that does not express these genes, were used as a negative control.


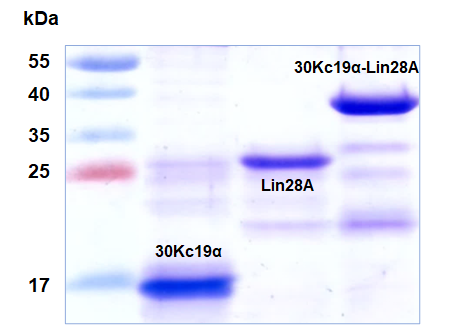


**Supplementary Figure 2.** SDS-PAGE results for 30Kc19α, Lin28A and 30Kc19α-Lin28A purified by Ni-NTA affinity chromatography.


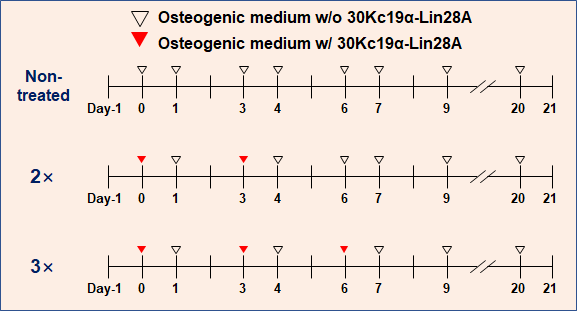


**Supplementary Figure 3.** Schedule for the treatments with 30Kc19α-Lin28A and osteogenesis of USCs.


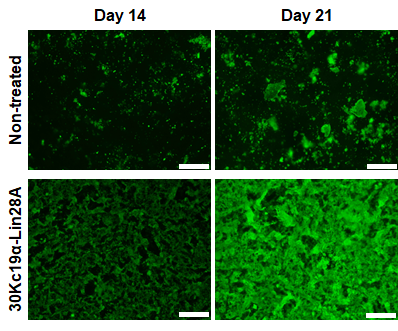


**Supplementary Figure 4.** Representative images of OsteoImage mineralization assay showing the formation of calcified matrices. On day 14 and 21 of osteogenesis, USCs (USC #1) were stained using fluorescent reagent and observed under a fluorescence microscope. Scale bar = 100 µm.


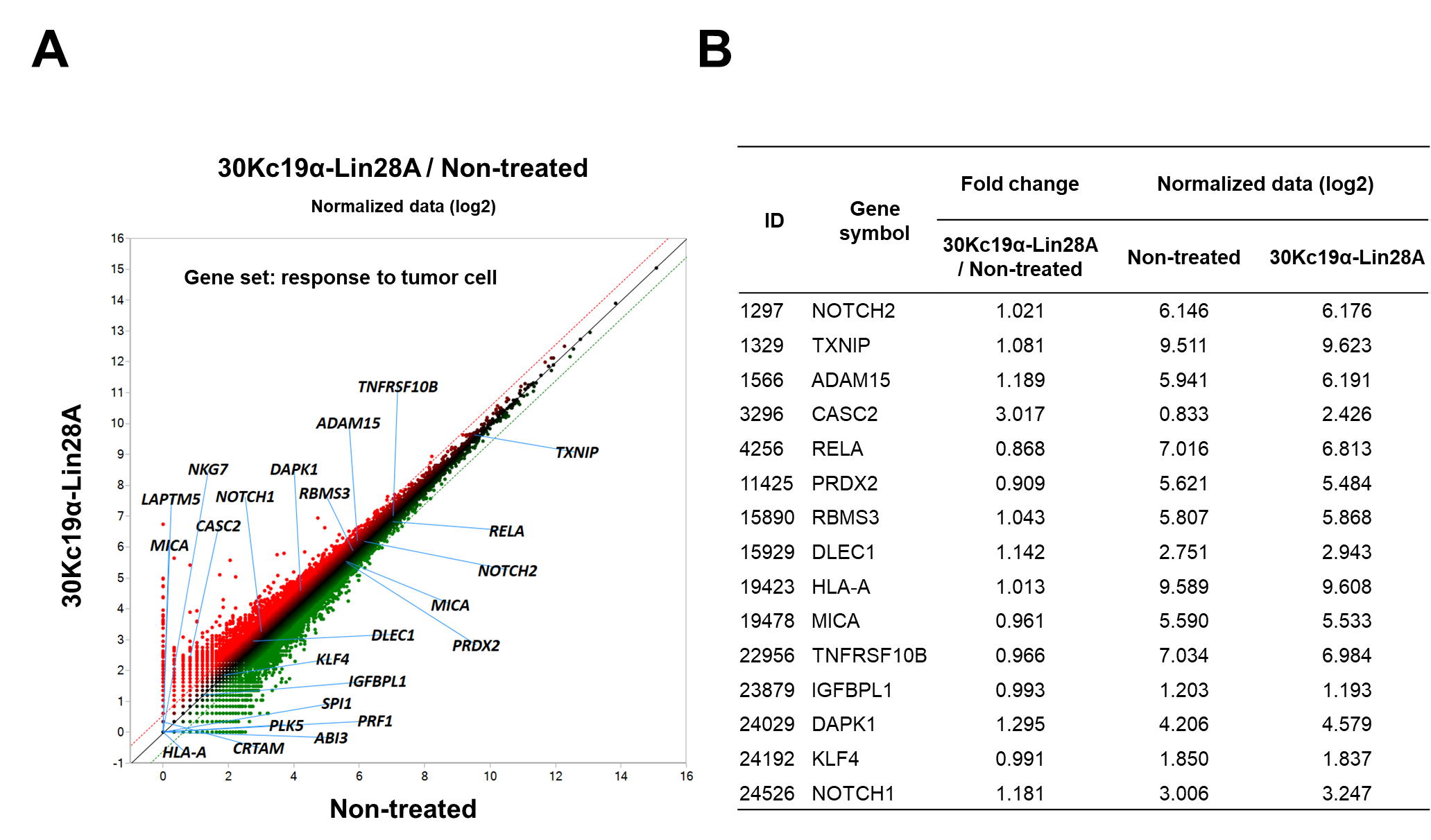


**Supplementary Figure 5.** RNA-seq analysis for a gene set related to tumor cell response. (A) Scatter plot illustrating the DEGs within the gene set associated with the response to tumor cells in 30Kc19α-Lin28A-treated USCs compared to non-treated USCs. (B) Comparison of the relative expression levels of each gene in non-treated and 30Kc19α-Lin28A-treated USCs.

## Supplementary Table

| Gene | Primer | Sequence (5’-3’) |
| --- | --- | --- |
| GAPDH | Sense | GTC AGT GGT GGA CCT GAC CT |
|  | Antisense | TGC TGT AGC CAA ATT CGT TG |
| Runx2 | Sense | GTC TTA CCC CTC CTA CCT GA |
|  | Antisense | TGC CTG GCT CTT CTT ACT GA |
| ALP | Sense | ACG TGG CTA AGA ATG TCA TC |
|  | Antisense | CTG GTA GGC GAT GTC CTT A |
| OPN | Sense | GTT TCG CAG ACC TGA CAT CC |
|  | Antisense | CAT TCA ACT CCT CGC TTT CC |
| OCN | Sense | CAA AGG TGC AGC CTT TGT GTC |
|  | Antisense | TCA CAG TCC GGA TTG AGC TCA |
| CD73 | Sense | CAG TAC CAG GGC ACT ATC TGG |
|  | Antisense | AGT GGC CCC TTT GCT TTA AT |
| CD90 | Sense | ATG AAC CTG GCC ATC AGC A |
|  | Antisense | GTG TGC TCA GGC ACC CC |
| CD105 | Sense | CCA CTA GCC AGG TCT CGA AG |
|  | Antisense | GAT GCA GGA AGA CAC TGC TG |

**Supplementary Table 1.** Primer list used for quantitative real‐time PCR.
